# COVID-19 Vaccine Hesitancy Among the Adult Population in Saudi Arabia

**DOI:** 10.7759/cureus.20197

**Published:** 2021-12-06

**Authors:** Ahmad M Alrajeh, Hanan Daghash, Shmayil F Buanz, Hanin A Altharman, Safia Belal

**Affiliations:** 1 College of Applied Medical Sciences, King Faisal University, Al-Ahsa, SAU; 2 Faculty of Medicine, University of Malaya, Kuala Lumpur, MYS; 3 Department of Nursing, College of Applied Medical Sciences, King Faisal University, Al-Ahsa, SAU

**Keywords:** saudi arabia, covid-19 vaccine perception, health belief model, covid-19 vaccine related conspiracy, covid-19 vaccine hesitancy

## Abstract

Background: Vaccine hesitancy in Saudi Arabia continues even after reaching 17 million doses. This study was conducted to comprehensively assess coronavirus disease 2019 (COVID-19) vaccine hesitancy in adult people who ignore the COVID-19 vaccine in the Saudi Arabian population and explore community awareness of public health after 17 million doses of COVID-19 vaccination.

Methods: A cross-sectional survey was used in this study. The questionnaire included three domains: demographic information, vaccine hesitancy by the health belief model related to the COVID-19 vaccine, and hesitancy by attitude and conspiracy towards the COVID-19 vaccine. A total of 401 adults participated in this study.

Results: The respondents’ perceptions of COVID-19 susceptibility and severity showed that the participants did not feel at risk nor believe that COVID-19 was serious. Connivance beliefs were found to be associated with reliance on social media as a major source of information about COVID-19 vaccines, and lack of trust in vaccine manufacturers (pharmaceutical companies). The majority of the respondents were concerned about the efficacy and safety of the COVID-19 vaccine, which can be reported as a major barrier to vaccination.

Recommendations: To increase vaccination rates, health authorities need to communicate both the benefits and risks of vaccination. In addition, we recommend using a qualitative study to understand and evaluate the participants' concepts in depth.

## Introduction

The world is witnessing a major global humanitarian crisis because of the spread of the coronavirus disease 2019 (COVID-19), which caused over 178,837,204 million cases and 3.8 million deaths [1]. The outbreak has triggered a global health crisis that has had a profound impact on the way we perceive the world and our daily lives. The crisis is not limited to disease control and crisis management but has the potential to have long-term and far-reaching consequences for states, societies, and international cooperation [2].

Governments around the world have taken precautionary measures such as social distancing, quarantine, and wearing masks to contain the spread of the disease and associated deaths, as well as the massive economic and social disruption worldwide [3]. To end the COVID-19 pandemic, the achievement of herd immunity is required, either through prior infection or vaccination [4]. An effective vaccine could be the optimal strategy to contain the spread of the COVID-19 and achieve positive clinical and socioeconomic outcomes.

The Saudi Ministry of Health has approved two COVID-19 vaccines developed by Pfizer/BioNTech and AstraZeneca for use in the Kingdom and 19 million doses have been administered already and are being used to immunize people in the country. The Ministry has clarified that vaccines play a crucial role in protecting against coronavirus infection and preventing deaths and hospitalizations due to infectious diseases. However, concerns about the efficiency and safety of COVID-19 vaccines are growing in Saudi society and around the world. These concerns are influenced by aspects such as convenience, comfort, confidence, and sociodemographic circumstances, and are caused by complicated, context-specific factors that change across time, place, and vaccines [5]. As a result, hesitancy in taking the vaccine was noted. According to the World Health Organization, vaccine hesitancy is defined as a “delay in acceptance or refusal of safe vaccines despite availability of vaccine services” [5].

Vaccination hesitancy may also be related to misinformation and conspiracy theories that are often spread online, especially on social media [6]. In addition, the structural factors of health inequalities, socioeconomic disadvantage, systemic racism, and barriers to access also contribute to vaccine skepticism and low vaccination rates. Although the term vaccine hesitancy is widely used, it might not accurately reflect these broader determinants that influence decisions to delay or refuse vaccination.

In addition, under the new rules, vaccination against coronavirus will be compulsory from August 1, 2021, for male and female workers in the workplace in all sectors (public, private, and non-profit) and for anyone wishing to take part in social, economic, commercial, cultural, scientific, entertainment, or sporting events. Moreover, our study can assist us and the competent authorities in identifying and clarifying the misconceptions that many people have about our study and addressing them promptly. Even though the current study will help policymakers develop messages to counteract an anti-vaccination campaign. Moreover, there is a paucity of references conducted in Saudi Arabia to determine the level of hesitancy to administer the COVID-19 vaccine.

Aim of the study

This study was conducted to comprehensively assess the COVID-19 vaccine hesitancy in adult people who ignore the COVID-19 vaccine in the Saudi Arabian population.

Research questions

What are the Saudi Arabian populations’ perceptions of COVID-19 susceptibility and severity? From where does community awareness of public health regarding coronavirus vaccination come from?

## Materials and methods

Study design

This study used a cross-sectional descriptive design to achieve the objective of the study using a survey.

Population and setting

This study was conducted to assess the public perception of the COVID-19 vaccine and the reasons for hesitancy among adults in the Kingdom of Saudi Arabia (KSA). Data collection took place between June 2021 and July 2021.

Sample size and sampling procedure

The study was conducted in 12 districts within the different provinces of Saudi Arabia. The target population for this study consisted of self-selected participants, with the following inclusion criteria: males and females aged 18 years and above, all of them were Saudi citizens, with different levels of education, from urban and rural areas, and they voluntarily agreed to participate in the study. We calculated the required sample size based on the total population in Saudi Arabia using the Robert Mason equation. The total population of Saudi Arabia is 34.27 million [7]; therefore, the minimum sample size was set to 384 participants. A total of 401 adults aged 18 years and above were selected using a convenient sample.

Data collection tool

The data were collected using a survey, which was adapted from Almaghaslah et al. [8] and modified to fit the sample criteria. It was administered using Google Forms (Google, Mountain View, CA) and was distributed via different social media platforms, including WhatsApp (Menlo Park, CA) and Twitter (San Francisco, CA). The questionnaire consisted of three domains. The first domain includes 12 questions related to demographic information and it was a combination of open-ended questions and closed questions. The second domain includes 14 questions related to vaccine hesitancy by the health belief model related to the COVID-19 vaccine. The third domain covered 15 questions related to hesitancy by attitude and conspiracy towards the COVID-19 vaccine.

To examine vaccine hesitancy health beliefs related to the COVID-19 vaccine, 14 items were generated using a four-point Likert scale (4 - strongly agree, 3 - agree, 2 - disagree, and 1 - strongly disagree). For the third part, a 15-item scale to examine and analyze vaccine hesitancy by attitude and conspiracy towards COVID-19 vaccine of participants was generated using a five-point Likert scale (5 - strongly agree, 3 - agree, 2 - neutral, 1 - disagree, and 0 - strongly disagree). The survey was originally written in the English language and was translated into the Arabic language to fit the sample criteria. Validity was verified by retranslating the Arabic language version of the questionnaire into the English language by writers who were bilingual speakers of both English and Arabic to ensure that the original meaning of the questions was maintained. The survey was distributed in both Arabic and English. Data were collected within one month, and each participant took 10-15 minutes to fill in the survey.

Ethical consideration

Before progressing to the survey, participants were informed about the study’s aims, advised of their ability to withdraw from the study at any time without providing a reason, and assured that the information and opinions provided would be anonymous and confidential. Ethical approval was obtained from the Research Ethics Committee of King Faisal University (KFU-REC/2021-06-43) viewing that the participants will not be exposed to any risks during the study. To ensure paradigm validity and consistency of the survey, a pilot sample of 20 participants was selected from the target population and was excluded from the total sample.

## Results

Among the 401 responses, more than 60% of respondents (63.6%) were identified as females. In the study, 38.7% of respondents were between the ages of 28 and 37 years. Just over half of the respondents in this study were married (51.4%), and over half were unemployed (53.9%). The survey found that more than 60% of the respondents held a university degree (62.8%), and over three-quarters of them lived in an urban area (75.1%). There was a relatively low number of participants who had chronic health problems (14.5%). Most participants (82.8%) had never experienced COVID-19 before. Most respondents rated their health status as good or quite good (74.6%). The majority of respondents (82.3%) stated that they would take precautions rather than get vaccinated. A more detailed description of this sample is provided in Table 1.

**Table 1:** Demographic characteristics of the study population (N = 401). COVID-19, coronavirus disease 2019.

		Number	Percent (%)
Gender	Male	146	36.4%
	Female	255	63.6%
Age	18 - 27	140	34.9%
	28 - 37	155	38.7%
	38 - 47	80	20.0%
	48 - 57	25	6.2%
	58 - 67	1	.2%
Social status	Married	206	51.4%
	Single	185	46.1%
	Divorced	10	2.5%
Educational level	High school	104	25.9%
	University student	252	62.8%
	Postgraduate student	32	8.0%
	Doctorate degree	12	3.0%
	Associate degree	1	.2%
Occupation	Employee	185	46.1%
	Unemployed	216	53.9%
Where do you live	Urban	301	75.1%
	Rural	100	24.9%
Which region	Riyadh	105	26.2%
	Eastern province	116	28.9%
	Makkah	75	18.7%
	Madinah	23	5.7%
	Asir	15	3.7%
	Al Baha	3	.7%
	Al Jawf	8	2.0%
	Al Qassim	14	3.5%
	Hail	8	2.0%
	Tabuk	28	7.0%
	Najran	2	.5%
	Jizan	4	1.0%
Do you have a chronic disease?	Yes	58	14.5%
	No	343	85.5%
How do you rate your overall health?	Very good	299	74.6%
	Good	79	19.7%
	Fair	17	4.2%
	Poor	5	1.2%
	Very poor	1	.2%
Ever have experience with COVID-19?	Yes	69	17.2%
	No	332	82.8%
Would you rather adhere to the precautions than take the vaccine?	Yes	330	82.3%
	No	71	17.7%
I will not take the vaccine until I am sure of its effectiveness in those around me.	Yes	233	58.1%
	No	168	41.9%

Table 2 presents descriptive information about the components of the health belief model. Respondents’ perceptions of COVID-19 susceptibility indicate that approximately 47.4% of respondents did not anticipate contracting COVID-19 within the next few months. According to the perception of COVID-19 severity, 30% of the respondents felt that the complications were not too severe (36.4%). Additionally, almost half of them are not afraid of becoming infected with COVID-19. More than 60% of respondents surveyed did not perceive the vaccination as having any benefit in reducing the chances of catching COVID-19 and reducing complications. The majority of respondents (70%) were concerned about the side effects of the vaccine and how they would interfere with normal daily activities, as well as concerns about vaccine efficacy (69.8%), vaccine safety (74.3%), and false vaccination with COVID-19 (66.6%). Based on the cause to action, around 14% of respondents refuse to take vaccines, even though much of the public does so.

**Table 2:** A descriptive analysis of hesitancy by health belief model toward COVID-19. COVID-19, coronavirus disease 2019.

	Strong agree	Agree	Disagree	Strongly disagree
Perceived susceptibility of contracting COVID-19				
My chance of getting COVID-19 in the next few months is great	13 (3.24%)	44 (10.97%)	154 (38.40%)	190 (47.38%)
I am worried about the likelihood of getting COVID-19	22 (5.50%)	42 (10.50%)	116 (29.00%)	220 (55.00%)
Getting COVID-19 is currently a possibility for me	14 (3.49%)	157 (39.15%)	94 (23.44%)	136 (33.92%)
Perceived severity				
Complications from COVID-19 are serious	34 (8.48%)	83 (20.70%)	138 (34.41%)	146 (36.41%)
I will be very sick if I get COVID-19	28 (6.98%)	65 (16.21%)	157 (39.15%)	151 (37.66%)
I am afraid of getting COVID-19	24 (5.99%)	56 (13.97%)	124 (30.92%)	197 (49.13%)
Perceived benefits of COVID-19 vaccination				
Vaccination is a good idea because I feel less worried about catching COVID-19	42 (10.47%)	36 (8.98%)	67 (16.71%)	256 (63.84%)
Vaccination decreases my chance of getting COVID-19 or its complications	42 (10.50%)	41 (10.25%)	71 (17.75%)	246 (61.50%)
Perceived barriers of COVID-19 vaccination				
I worry the possible side-effects of COVID-19 vaccination would interfere with my usual activities	280 (70.00%)	62 (15.50%)	30 (7.50%)	28 (7.00%)
I am concerned about the efficacy of the COVID-19 vaccination	279 (69.75%)	57 (14.25%)	39 (9.75%)	25 (6.25%)
I am concerned about the safety of the COVID-19 vaccination	297 (74.25%)	45 (11.25%)	40 (10.00%)	18 (4.50%)
I am concerned about the faulty/fake COVID-19 vaccine	264 (66.00%)	57 (14.25%)	53 (13.25%)	26 (6.50%)
Cues to action				
I will only take the COVID-19 vaccine if I was given adequate information about it	59 (14.86%)	88 (22.17%)	106 (26.70%)	144 (36.27%)
I will only take the COVID-19 vaccine if the vaccine is taken by many in the public	21 (5.25%)	42 (10.50%)	122 (30.50%)	215 (53.75%)

Table 3 presents the perception of hesitancy regarding the COVID-19 vaccine based on attitudes toward the vaccine and belief in conspiracy theories. Most respondents (around 80%) were not comfortable with the COVID-19 vaccine and believed that it would probably not be able to protect them. Moreover, the majority of respondents strongly believed that coronaviruses are a myth that is imposed on people to force vaccination and that people are misled about the efficacy of vaccines. Furthermore, the majority of respondents believe that the pharmaceutical industry is encouraging coronavirus spread to make a profit through vaccination, and the pharmaceutical industry covers up the side effects of vaccines. Three-quarters of respondents rated countries that manufacture vaccines neutrally, regardless of whether they were located in America, Europe, Russia, China, or India. Therefore, their opinions are not influenced by the country that manufactures the vaccine.

**Table 3:** A descriptive analysis of vaccine hesitancy by attitude and conspiracy towards COVID-19 vaccine. COVID-19, coronavirus disease 2019.

	Strong agree	Agree	Neutral	Disagree	Strongly disagree
I think the COVID-19 vaccine probably will not work	238 (59.50%)	49 (12.25%)	59 (14.75%)	24 (6.00%)	30 (7.50%)
I do not trust the COVID-19 vaccine	273 (68.42%)	39 (9.77%)	41 (10.28%)	19 (4.76%)	27 (6.77%)
I think the COVID-19 vaccine is unnecessary	258 (64.50%)	41 (10.25%)	40 (10.00%)	40 (10.00%)	21 (5.25%)
I think it is not important to get a vaccine to protect people from the COVID-19	230 (57.79%)	45 (11.31%)	52 (13.07%)	39 (9.80%)	32 (8.04%)
I do not need a COVID-19 vaccine because I am healthy and at low risk for infection	239 (60.05%)	60 (15.08%)	40 (10.05%)	35 (8.79%)	24 (6.03%)
I do not need a COVID-19 vaccine because even if I get infected, I will not become seriously ill	205 (51.38%)	73 (18.30%)	53 (13.28%)	37 (9.27%)	31 (7.77%)
Pharmaceutical companies are encouraging the spread of coronavirus to make a profit through selling vaccine	230 (57.50%)	59 (14.75%)	62 (15.50%)	29 (7.25%)	20 (5.00%)
The coronavirus is a myth to force vaccinations on people	175 (43.97%)	46 (11.56%)	75 (18.84%)	62 (15.58%)	40 (10.05%)
Drug companies cover up the side effects of vaccines	245 (61.40%)	65 (16.29%)	37 (9.27%)	31 (7.77%)	21 (5.26%)
People are deceived about the effectiveness of vaccines	219 (54.89%)	71 (17.79%)	62 (15.54%)	30 (7.52%)	17 (4.26%)
COVID-19 vaccine can result in autism	82 (20.55%)	36 (9.02%)	174 (43.61%)	61 (15.29%)	46 (11.53%)
A coronavirus vaccination could give one coronavirus	140 (35.09%)	56 (14.04%)	119 (29.82%)	49 (12.28%)	35 (8.77%)
COVID-19 vaccines made in America and Europe are safer than those made in other countries	17 (4.26%)	29 (7.27%)	138 (34.59%)	79 (19.80%)	136 (34.09%)
COVID-19 vaccines made in China and Russia are safer than those made in other countries	7 (1.76%)	18 (4.52%)	156 (39.20%)	84 (21.11%)	133 (33.42%)
COVID-19 vaccines made in India are safer than those made in other countries	3 (0.75%)	3 (0.75%)	165 (41.46%)	91 (22.86%)	136 (34.17%)

Using binomial logistic regression, we tested the impact of health beliefs, attitudes, and conspiracy toward the COVID-19 vaccine on predicting hesitancy toward the vaccine. The logistic regression model was statistically significant (χ2 (7) = 255.464, p < 0.001), it explained 75.3% (Nagelkerke R^2^) of the variance, and was able to correctly identify 80% of cases. Among the five predictor variables, only three were statistically significant: perceived susceptibility to contracting COVID-19, perceived benefits of COVID-19 vaccination, and perceived barriers to the vaccination of COVID-19. Perceived susceptibility to contracting COVID-19 and worry about the likelihood of contracting COVID-19 (OR = 0.261, 95% CI 0.106-0.639), and the perception that vaccination is a good idea to reduce the worry about catching COVID-19 under the perceived benefit construct (OR = 0.286, 95% CI 0.124-0.660) were significant predictors of hesitancy to vaccinate. Thus, increases in the perception of susceptible contrast and perception of the benefits of vaccines were associated with decreases in the odds ratio of hesitation toward vaccines. This means that those participants who felt more confident about the benefits of vaccines and the severity of contracting diseases showed less hesitation toward vaccines. According to this study, health authorities need to communicate both the benefits and risks associated with vaccinations to increase vaccination rates. According to this study, perceived barriers to vaccination were a significant predictor of hesitancy (OR = 2.562, 95% CI 1.117-5.8780), as every increase in perceived barriers toward COVID-19 vaccine increased the odds of refusing vaccine by two times. Therefore, people who were concerned about the safety and risks associated with vaccinations were more likely to avoid vaccinations (Table 4).

**Table 4:** Predictors of vaccine hesitancy for COVID-19 based on logistic regression (N = 401). COVID-19, coronavirus disease 2019; SE, standard error.

	B	SE	Wald	Odds ratio	95% CI for odds ratio	
					Lower	Upper
Perceived susceptibility of contracting COVID-19	−1.344	.457	8.631	.261	.106	.639
Perceived severity	−.484	.413	1.377	.616	.274	1.384
Perceived benefits of COVID-19 vaccination	−1.250	.426	8.616	.286	.124	.660
Perceived barriers of COVID-19 vaccination	.941	.424	4.932	2.562	1.117	5.878
Cues to action	.048	.357	.018	1.049	.521	2.114
Attitude towards COVID-19 vaccine	.756	.454	2.770	2.129	.874	5.186
COVID-19 vaccine conspiracy	−.752	.502	2.249	.471	.176	1.260
Constant	5.843	2.189	7.125	344.924		

**Figure 1:**
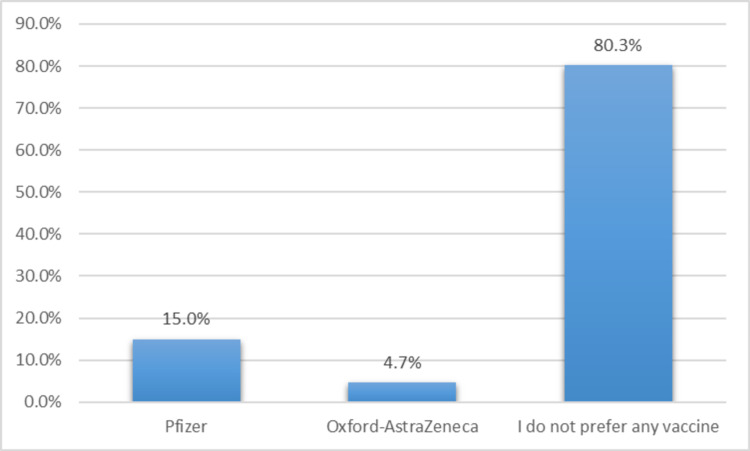
Types of COVID-19 vaccine preference among respondents. COVID-19, coronavirus disease 2019.

Figure 1 shows that 80.3% of respondents were not interested in any type of COVID-19 vaccine, while 15% preferred the Pfizer-BioNTech vaccine and 4.7% preferred the Oxford-AstraZeneca vaccine.

## Discussion

The government of Saudi Arabia urges its residents to receive the COVID-19 vaccine since the number of doses has reached 17 million, but some residents refuse to receive the vaccine despite its safety, reliability, and widespread acceptance.

Health belief model

Based on the information about the components of the health belief model, perceived susceptibility, and severity, 47.38% of respondents strongly disagree about their chance of getting COVID-19 in the next few months, 55.00% did not worry about getting COVID-19, 36.41% said that the complications from COVID-19 were not serious, and 49.13% were not afraid of getting COVID-19. It could be interpreted that the respondents are not feeling at risk and not believing that COVID-19 is serious perhaps because they believe that their self-immunity is sufficient or adherence to precautionary measures is sufficient for them, as 82.3% of respondents prefer to adhere to the precautions than take the vaccine, and 60.05% said that they did not need a COVID-19 vaccine because they were healthy and at low risk for infection.

In a previous study conducted in Saudi Arabia [8], 29.5% of respondents believed that COVID-19 and its complications are serious, while 13.8% disagree and 5.4% strongly disagree. The reason for this opposite finding is that previous studies included both opponents and supporters of the vaccine, and it is possible that the participation of supporters is more than that of opponents, unlike this study, which included only opponents. However, previous studies have also shown that there is a high level of hesitancy toward vaccination [8].

In terms of perceived benefits and perceived barriers to COVID-19 vaccination, 63.84% of respondents strongly disagree that vaccination is a good idea, 61.50% strongly disagree that vaccination decreases their chance of getting COVID-19 or its complications, 70.00% worry about the side effects of vaccination, 69.75% concern about efficacy, 74.25% concern about safety, and 66.00% concern about the faulty/fake COVID-19 vaccine. Consequently, the safety and efficacy of the vaccine are likely to cause people to refrain from vaccination if they are concerned. As stated in a previous study by Almaghaslah et al. [8], COVID-19 vaccine safety and efficacy are the major barriers to vaccination.

The World Health Organization has clarified the effectiveness of the vaccine as it prevents people from getting seriously ill or dying from COVID-19 [9]. One of the reasons for their concern about the effectiveness and safety of the vaccine is that it does not prevent infection with COVID-19 after the two doses. One of the studies conducted in Saudi Arabia entitled “Safety and Reactogenicity of the ChAdOx1 (AZD1222) COVID-19 Vaccine in Saudi Arabia” [9] reported that no major side effects have been reported from ChAdOx1 and no breakthrough infection during the observation period was noted. Another study conducted on April 30, 2021 in the United States by Birhane et al. [10] reported that 101 million people have been fully vaccinated against COVID-19. However, severe acute respiratory syndrome coronavirus 2 (SARS-CoV-2) transmission was widespread during the observation period in many parts of the country. Despite the high quality of FDA-approved vaccines, a breakout may be expected, especially before an immunity level reaches a point where it can reduce transmission. The COVID-19 cases represent a small percentage. In Israel, the incidence of SARS-CoV-2 outcomes decreased in all age categories [11].

If herd immunity is not achieved, a breakthrough infection is possible. The above studies have shown that the number of breakthrough cases decreases with an increase in the number of vaccinators.

Vaccine hesitancy by attitude and conspiracy towards COVID-19 vaccine

The problem with COVID-19 is that people are now exposed to it for the first time, as no one has acquired immunity against COVID-19. Of the participants, 64.50% thought that the COVID-19 vaccine was unnecessary, 60.05% thought that they did not need a COVID-19 vaccine because they were healthy and at low risk for infection, and 51.38% thought that they do not need a COVID-19 vaccine because even if they get infected, they will not become seriously ill. On July 14, 2021, WHO reported that vaccines work by instructing and preparing the immune system to recognize and fight off viruses and bacteria. When the body is later exposed to disease-causing germs after vaccination, the body is immediately ready to destroy them, preventing illness [12]. In general, COVID-19 vaccines are safe for most people older than 18 years who do not have pre-existing conditions, including autoimmune disorders. These conditions include hypertension, diabetes, asthma, pulmonary, liver, and kidney disease, as well as chronic infections that are stable and controlled [12]. In addition, everyone needs to take the vaccine to reduce the infection and spread of COVID-19.

As for the conspiracy idea, in both populations in Ireland and the United Kingdom [13], it was found that those who reject COVID-19 were less likely to obtain information about the pandemic from traditional sources and had a higher level of mistrust for such sources than respondents who accepted the vaccine. Of the respondents, 57.50% said that pharmaceutical companies are encouraging the spread of coronavirus to make a profit through selling vaccine, 43.97% said that the coronavirus is a myth to force vaccinations on people, and 61.40% said that drug companies cover up the side effects of vaccines. All these thoughts are related to the idea of conspiracy. In a previous study among the public in Jordan, Kuwait, and other Arab countries [14], one of the aims was to assess the association between COVID-19 vaccine acceptance and conspiracy beliefs.

According to the current study, using social media as a major source of information regarding COVID-19 vaccinations leads to vaccine hesitancy. Additionally, conspiracy theories could be influenced by distrust of the companies that manufacture vaccines (pharmaceutical companies) and the healthcare professionals.

Community awareness of public health after 17 million doses

The Saudi Ministry of Health is working hard to clarify the information needed to identify COVID-19 and the vaccines available in the Kingdom. It allocated a special website of the Ministry of Health in the name of COVID-19 awareness [15], which has been translated into several languages. In addition, it stressed the need to take the vaccine from August 1 for male and female workers in the workplace in all sectors (public, private, non-profit) and for anyone wishing to take part in a social, economic, commercial, cultural, scientific, entertainment, or sporting event.

Therefore, the number of people coming to vaccination centers in all regions of the Kingdom increased, but 82.3% of the respondents preferred to adhere to the precautions than take the vaccine.

Limitations of the study

Considering the current study, there are some limitations to take into account. Because the study used a non-probability sample, we should be cautious about generalizing the results. To some extent, it is difficult to compare the data from this study with national data. This is because the distribution of respondents by age, gender, region, residence, occupation, family structure, and education does not consistently reflect the national population. In addition, the self-reported data from this study may be biased to some degree due to reporting bias. Because the data were collected online, they were self-reported, so not all groups of people were included in this study, such as illiterate people who do not have access to social networks. As a final note, this study was designed as a cross-sectional study where causality is limited.

## Conclusions

The Kingdom of Saudi Arabia encourages residents to be vaccinated against COVID-19 to maintain community immunity. Some residents refused the vaccine, even though its safety, reliability, and effectiveness were confirmed. It is more common for people to refuse vaccinations if they are concerned about their safety and effectiveness. People who were aware of the benefits of vaccination and the severity of the disease were less hesitant to get vaccinated. To increase vaccination rates, health authorities need to communicate both the advantages and risks of vaccination. Further, we recommend using a qualitative study to get a better understanding of the participants' responses.
